# Molecular Mechanisms of Negative Effect of Systemic Lupus Erythematosus on Oogenesis and Meiotic Processes in Oocytes

**DOI:** 10.3390/ijms27093993

**Published:** 2026-04-29

**Authors:** Stefka Delimitreva, Ana Blagoeva, Irina Chakarova

**Affiliations:** Department of Biology, Medical University of Sofia, 2 Zdrave Street, 1431 Sofia, Bulgaria; 114000@students.mu-sofia.bg (A.B.); i.chakarova@medfac.mu-sofia.bg (I.C.)

**Keywords:** oogenesis, oocyte, meiosis, systemic lupus erythematosus (SLE)

## Abstract

Systemic lupus erythematosus is an autoimmune disease that mainly affects women of reproductive age. Its pathological manifestations directly and indirectly negatively affect the ovarian reserve, the quality of oocytes, and the precise mechanisms of meiosis. This review presents the molecular mechanisms by which lupus damages ovarian tissue and meiosis in oocytes. The role of chronic inflammation, impaired hormonal levels, and the presence of specific autoantibodies are considered. The available data on how oocyte structures (meiotic spindle, actin cytoskeleton, membrane organelles and chromatin) are damaged by lupus symptoms are summarized.

## 1. Introduction

Systemic lupus erythematosus (SLE), often referred to simply as lupus, is a chronic autoimmune disease in which the body’s immune system mistakenly attacks its own healthy tissues and organs. This leads to broad-spectrum inflammation that can affect almost any part of the body.

Although lupus does not directly cause infertility, the disease reduces fertility through several main mechanisms affecting both women and men. In women, the three main causes can be summarized as follows: a decrease in ovarian reserve [1]; low levels of anti-Müllerian hormone (AMH), which is a marker of oocyte count [2]; autoimmune oophoritis [3]. In men, lupus reduces fertility by: damage to the testicles as a result of inflammation affecting the seminal ducts, which leads to a decrease in sperm count and motility; erectile dysfunction as a result of vascular problems or side effects of medications [4].

Although lupus can lead to complete infertility in both sexes (especially when applying aggressive treatment), the disease affects fertility more noticeably in women. The first reason women are more affected is the fact that lupus often occurs at the peak of their reproductive age (15–45 years) [5]. In addition, autoimmune oophoritis, which leads to abnormally low levels of AMH, directly reduces ovarian reserve. Even with successful conception, the risk of fetal loss is significantly higher due to the presence of specific autoantibodies [6].

The pathologic processes typical for systemic lupus can negatively affect ovarian reserve even in patients who have not taken high-risk medications such as cyclophosphamide [7]. In this review, we look at how the manifestations of systemic lupus disease itself reduce female fertility, damaging oocyte meiosis. This could be the result of both direct damage to the oocyte structures and indirect change of the microenvironment of the follicle, in which the meiotic maturation of the oocyte takes place. Generally speaking, lupus affects oogenesis through damage to the reproductive organs and systemic disorders that result from chronic inflammation and an autoimmune attack.

## 2. What Are the Molecular Mechanisms of Chronic Inflammation That Alter Hormonal Levels in Women with Lupus

The general condition of chronic inflammation associated with lupus can disrupt the hypothalamic–pituitary–ovarian (HPO) axis [8]. This is carried out through complex interactions between the immune and endocrine systems. It has been proven that cytokines are specifically involved in the process. Pro-inflammatory cytokines such as IL-1, IL-6, and TNF-α, whose levels are elevated in lupus, directly inhibit the secretion of gonadotropin-releasing hormone (GnRH) in the hypothalamus and of luteinizing hormone (LH) and follicle-stimulating hormone (FSH) in the pituitary gland [9].

**The three main pro-inflammatory cytokines inhibit GnRH secretion**. GnRH neurons (gonadotropin-releasing hormone neurons) are the “conductors” of the reproductive system in the human body. These are a small group of specialized nerve cells in the hypothalamus that secrete the decapeptide GnRH. The mentioned cytokines IL-1, IL-6, and TNF-α activate inhibitory neurotransmitters. Thus, they fail to stimulate the secretion of GnRH by GnRH neurons. Binding of IL-1 to its receptor type I (IL-1RI) on GnRH neurons triggers a cascade of intracellular signals that directly attack both the hormone’s gene transcription and the stability of its mRNA. In addition, the transport of GnRH along the axons to the median eminence, where it is released into the bloodstream, is blocked [10]. Unlike interleukin-1 (IL-1), whose inhibitory action is direct, the role of interleukin-6 (IL-6) in the secretion of GnRH is indirect—IL-6 modulates the neural networks that control GnRH neurons. IL-6 triggers the release of corticotropin-releasing hormone (CRH) in the hypothalamus, which leads to suppression of the pulse activity of GnRH neurons [11]. This is the main mechanism by which IL-6 indirectly shuts down reproductive function during illness or stress. TNF-α suppresses GnRH secretion through mechanisms that overlap with those of IL-1, but with a specific focus on glial cells and oxidative stress in the hypothalamus. Like IL-1, TNF-α binds to its receptors (TNFR1) on GnRH neurons and activates the signaling function of NF-κB (nuclear factor kapa-B). NF-κB can directly or indirectly suppress the promoter activity of the GnRH gene, resulting in less mRNA production, as well as a shorter mRNA half-life for GnRH [12]. In addition, increased concentrations of TNF-α induce apoptosis in hypothalamic cells and disrupt the electrophysiological activity of neurons [13].

Cytokines also act through endogenous opioid peptides (endorphins and dynorphins). IL-1, IL-6, and TNF-α can cross the blood–brain barrier in areas such as circumventricular organs or be produced locally by microglia [14]. They activate signaling pathways that stimulate the gene expression of opioid peptide precursors and thus encourage their production. These “natural painkillers” bind to receptors on GnRH neurons and directly reduce the frequency of their secretion [15]. Since GnRH must be secreted at precise intervals (pulsations), even a slight disturbance in the rhythm of pulsations caused by these cytokines is enough to prevent the peak of LH, resulting in a lack of ovulation.

Inflammatory mediators enhance the signaling of GABA (gamma-aminobutyric acid), which acts as the main inhibitory neurotransmitter for the reproductive axis [16]. This is based on inappropriate activation of GABA-A receptors, which are ligand-dependent chloride channels [17]. Their activation leads to the entry of chloride ions into the cell (hyperpolarization of the neuron), which suppresses the electrical activity of GnRH neurons. This reduces the secretion of GnRH and LH and FSH, respectively, which can lead to a delay in sexual development and secondary hypogonadism. At first glance, this contradicts the fact that systemic lupus is usually associated with an increased level of FSH. The explanation is as follows: before the onset of puberty, GABA levels in the hypothalamus are high, which keeps the reproductive system “off” by suppressing GnRH; if this inhibitory activity does not decrease in the normal timeframe, it leads to low levels of FSH and LH, which disturbs sexual development [18]. In the mature female body, under conditions of high stress or severe metabolic changes (such as those in lupus), GABA levels can be abnormal [19]. During times of physiological stress, inappropriately high GABA activity on GnRH neurons may lead to secondary hypogonadism, especially when the disease has already damaged the ovaries.

Another mechanism by which pro-inflammatory cytokines effectively disrupt the function of GnRH neurons is through the kisspeptin system. Kisspeptin is a protein that acts as a major trigger for the onset of puberty and reaching reproductive maturity. It performs its function by stimulating GnRH neurons [20]. IL-1 and TNF-α significantly reduce the levels of kisspeptin in the nuclei of the hypothalamus due to direct suppression of the expression of its gene Kiss1. Cytokines have been shown to reduce the levels of kisspeptin mRNA in the hypothalamus (especially in the arcuate nucleus) [15]. Without enough kisspeptin, GnRH neurons remain “silent” even if all other ovulation conditions are present [21].

Hormonal disorders in women with lupus are not only a consequence of the disease, but also actively support its manifestations. This creates a vicious circle that significantly affects the chance of conception and a normal pregnancy. This vicious circle is driven by the following facts. (1) In patients with lupus, the level of certain estrogen metabolites (such as 16α-hydroxyestrone) is often increased, which have a strong immune-boosting effect and supports inflammation [22]. (2) Inflammation is associated with reduced levels of protective androgens such as testosterone and DHEA (dehydroepiandrosterone) [23]. DHEA generally helps regulate the immune response, and its deficiency can worsen disease activity. (3) Women with SLE often have lower levels of progesterone, which deprives them of its natural immunoregulatory benefits [24]. Progesterone inhibits the activation of T cells and the production of pro-inflammatory cytokines. When its level is low, the immune system becomes more aggressive [25]. (4) Women with lupus often have higher levels of FSH and prolactin, but lower levels of LH and progesterone, which can disturb the ovulation signals [26].

**The activity of GnRH neurons is controlled by microglia.** Microglial cells are responsible for the physical formation of neural networks—they can phagocytize excess synapses or help build new synapses around GnRH neurons [27]. Thus, before puberty, the microglia are responsible for establishing the correct density of the connections, which is critical for the activation of the reproductive axis. In sexually mature organisms, the action of microglia allows the brain to adapt the frequency of GnRH impulses to external signals. Microglia communicate with neurons through chemical signals. In the case of systemic stress or infection, microglia secrete molecules such as TNF α and IL-1β [15]. They usually have an inhibitory effect on GnRH neurons, which explains why reproductive function often stops during severe illness. Microglial cells send signals to astrocytes, which in turn physically regulate the access of GnRH neurons to blood vessels in the eminentia mediana [28]. Thus, the microglia indirectly control how much GnRH will reach the pituitary gland.

In SLE, the dialogue between microglia and GnRH neurons is disrupted by three main mechanisms. (1) Systemic inflammation and specific autoantibodies damage the blood–brain barrier [15]. This allows pro-inflammatory factors and immune cells to enter the hypothalamus. As a result, the microglia go from a support state (homeostatic) to an aggressively activated state. (2) In the vicinity of GnRH neurons, lupus-activated microglia secrete large amounts of pro-inflammatory cytokines (including IL-6 and TNF-α) [29]. These signaling molecules suppress the gene expression of GnRH and the frequency of its pulses [30]. In addition, activated microglia secrete reactive oxygen species (ROS). They damage the mitochondria of GnRH-neurons, making them less responsive to stimulation by kisspeptin. (3) In lupus, hyperactivated microglia mistakenly attack and remove synaptic connections between kisspeptin neurons and GnRH neurons [31]. Thus, the communication chain is physically interrupted, leading to hypogonadotropic hypogonadism (low levels of LH and FSH, although the ovaries may be healthy).

**Lupus increases the production of 16α-hydroxyestron**. Pro-inflammatory cytokines (especially IL-6) modulate the activity of enzymes in the liver. In lupus, the activity of the enzyme CYP1A1/CYP3A4 is increased. This enzyme directs metabolism toward the synthesis of 16α-hydroxyestron. On the other hand, the activity of the enzyme CYP1A2 is reduced. It produces the “good” 2-hydroxyestrone [32]. This is due to the binding of IL-6 to its receptor on the surface of hepatocytes, resulting in the activation of the STAT3 protein. This suppresses the activity of key nuclear receptors (PXR for CYP3A4 and AhR for CYP1A1), which are responsible for the transcription of genes for the liver enzymes above-mentioned [33].

While normal estradiol binds to receptors temporarily and is then released, 16α-hydroxyestron forms a stable covalent bond (via a Schiff base) with estrogen receptors [34]. This provides long-lasting and powerful stimulation of the immune system. 16α-Hydroxyestron also binds covalently to other cellular proteins. An important fact is that 16α-hydroxyestron is very prone to form covalent bonds with histone H1. Thus, H1 and 16α-hydroxyestron form structures called adducts [22]. The immune system recognizes these adducts as foreign and activates B cells to produce high-affinity autoantibodies [35], which directly increases disease activity.

**In women with lupus, chronic inflammation is directly related to lower levels of testosterone and DHEA**. This condition is often referred to as “androgen deficiency” and plays a key role in the course of the disease [36]. Inflammatory processes in lupus suppress androgens through several specific pathways. Severe systemic inflammation leads to a redirection of hormone production in the adrenal glands—they prioritize producing cortisol, which suppresses inflammation, but this takes resources away from the DHEA synthesis pathway [23]. In addition, in women with lupus, there is an accelerated oxidation of testosterone, which leads to its faster depletion in the blood. Oxidation occurs primarily at the C-17 position of the testosterone molecule. This process transforms testosterone into weaker androgens such as androstenedione, which can then be easily converted into estrogens (such as estrone and estradiol) by the enzyme aromatase [37]. Low testosterone levels are also caused by some defects in enzymes responsible for converting precursors to DHEA (e.g., 17,20-lyase). Such enzyme defects are often seen in patients with lupus [38].

DHEA is not just a sex hormone; it acts as an important immunomodulator. Its deficiency leads to several adverse effects. Insufficient DHEA production leads to an imbalance of T-helpers—DHEA directs the immune response to the Th2-type, which stimulates B cells to produce more autoantibodies. DHEA is known to act as a natural transcriptional activator for the IL-2 gene in CD4+ T cells [39]. In lupus, IL-2 levels are often low due to DHEA deficiency. This is another reason why DHEA deficiency impairs the function of T cells [40]. Eventually, it comes to a situation defined as “estrogen dominance”: androgens (such as DHEA) have a protective effect against autoimmune processes and therefore their deficiency encourages the expression of estrogens, which have an immune-stimulating effect and can exacerbate symptoms.

DHEA deficiency, in addition to stimulating the appearance of too much Th2, also leads to problems in the immune clearance of unnecessary and damaged cells [41]. DHEA stimulates the expression of specific activating receptors on the surface of Natural Killer (NK) cells. Low levels of DHEA are associated with the reduced cytotoxicity of NK cells, which are the first line of defense in recognizing infected or cancerous cells [42]. In DHEA deficiency, NK cells recognize their targets with less probability. In addition, DHEA acts as a natural counterbalance to cortisol [43]. With DHEA deficiency, the suppressory action of cortisol on the immune system remains uncontrolled, leading to persistent immunosuppression and difficulty clearing pathogens, including abnormal cells.

Low levels of IL-2 (result of low DHEA) lead to defects in the regulation of the level of activation-induced cell death (AICD) [44]. AICD is a mechanism that is responsible for blocking the overactive action of T-lymphocytes in response to repeated stimulation of their T-cell receptors (TCR). Thus, AICD helps maintain peripheral immune tolerance. When this control mechanism is disrupted, the autoreactive T cells that need to be eliminated survive and continue to drive the Th2-mediated autoimmune attack.

**Lupus leads to a decrease in progesterone synthesis**. In some patients with lupus, antibodies against the corpus luteum are produced [45]. Since the corpus luteum is the main source of progesterone after ovulation, its damage directly disrupts the production of the hormone. The accumulation of immune complexes in the ovaries causes tissue damage and the premature depletion of eggs, which is associated with general hormonal deficiency. Chronic inflammation also leads to such a result by disrupting the hypothalamic–pituitary–ovarian axis, as explained above.

**Low progesterone levels make the immune system more aggressive**. Progesterone acts as a regulator that calms the body’s defenses [46]. When its amount is reduced, estrogen dominance occurs (while progesterone suppresses inflammation, estrogen stimulates the immune response). Under normal conditions, progesterone blocks the activation of the factor NF-kB, which is responsible for the production of pro-inflammatory cytokines such as TNF-α and IL-6 [47]. With progesterone deficiency, the synthesis of these inflammatory signaling molecules increases. Progesterone is involved in maintaining a balance between T helper types. It encourages Th2 response (which is generally anti-inflammatory) and suppresses the Th1 and Th17 pathways, which are associated with aggressive immune processes [48]. In addition, with progesterone deficiency, not enough regulatory T-cells (Tregs) are produced, whose role is to prevent attacks on their own structures [49].

**Lupus increases the level of follicle-stimulating hormone**. An abnormal increase in FSH in women with lupus is considered a marker of premature ovarian failure. The link between systemic lupus and FSH levels is not a direct consequence of the disease itself, but is the result of its impact on ovarian reserve as a result of chronic inflammation and the action of autoantibodies [50].

**Lupus increases the level of prolactin**. Hyperprolactinemia is observed in about 20–30% of patients with systemic lupus [51]. This fact is based on the complex relationship between the immune and endocrine systems. Prolactin is not only produced by the pituitary gland. In patients with lupus, lymphocytes also begin to secrete prolactin [52]. In this context, it acts as a cytokine that stimulates inflammation and the immune response. In addition, the secretion of prolactin is suppressed by dopamine. In patients with SLE, defects in dopamine metabolism are often observed, which removes the “brake” on the production of prolactin [53].

In a normal state, anergic B cells are in a state of “sleep” because their threshold of response to self-antigens is very high. When prolactin binds to its receptor on the surface of the B-cell, the enzyme JAK2 (attached to the intracytoplasmic receptor domain) is activated. JAK2 triggers a change in cellular responses. As a result of this change, calcium is released from the endoplasmic reticulum. When calcium stores in the endoplasmic reticulum are depleted, the cell activates a mechanism called Store-Operated Calcium Entry (SOCE). Specific proteins (STIM1) sense the emptiness in the depots and signal the calcium channels in the plasma membrane (Orai1) to open [54]. This allows external calcium to enter the cell, maintaining high levels for a long time. This constant calcium background acts as an amplifier of B cell activation signals, which stimulates them to react against self-structures that they would otherwise ignore.

**In systemic lupus, LH levels are often lowered**. This is due to chronic inflammation and ovarian damage, which leads to premature depletion of ovarian reserve. Although this usually leads to an increase in LH (as a compensatory mechanism), in certain phases or with complex involvement of the hypothalamus, the balance can be disturbed [55]. It is interesting to note that in men with lupus, the opposite is often observed—increased levels of LH, which is a sign of the body’s attempt to compensate for low testosterone (hypoandrogenism) [4].

The reduced LH level is not a direct activator of lupus, but rather a key indicator of hormonal breakdown that removes the body’s protective barriers against inflammation. The main reason for this is a deficiency of protective progesterone [56]. With low LH and a lack of progesterone, even if the estrogen levels are normal, a state of estrogen dominance is created, which was commented on above. Low LH is also associated with lower levels of androgens (such as testosterone and DHEA), which in women suppresses inflammation. The connection is due to the fact that LH is a major conductor of hormonal production in the ovaries. LH binds to specific receptors in theca cells [57]. As a result, these cells produce androgens (testosterone and androstenedione). When LH levels are low, this signal is absent, and androgen production drops dramatically.

**Lupus reduces the level of anti-Müllerian hormone**. AMH is produced in the ovaries, more specifically in granulosa cells that surround and feed the immature oocytes. The hormone is synthesized in the preantral and small antral follicles. When a follicle becomes dominant and prepares for ovulation, the production of AMH in it stops. Since AMH is secreted only by these small reserve follicles, its level in the blood serves as a direct marker of ovarian reserve [58]. The smaller the number of preantral and small antral follicles, the lower the AMH level.

## 3. How Does Lupus Cause a Decrease in the Number of Preantral Follicles

As is widely known, the disease itself triggers immune response against ovarian tissue. It begins with the infiltration of T-lymphocytes into the ovary and the production of antiovarian antibodies, leading to chronic inflammation and apoptosis of the different types of cells in the ovary. In these processes, the function of Tregs, which normally suppress autoimmune attacks, is impaired [59]. In principle, abnormal invasion of T cells is the basis of the autoimmune process in systemic lupus. Impaired Tregs function allows effector T-lymphocytes to enter organs that are normally protected, including the ovaries. To understand how this happens in the ovary, we need to consider it as a place with a privileged immune status, which in lupus is impaired. The ovaries have a protective barrier that restricts the access of immune cells to the follicles [60]. With the abnormal action of Tregs, the production of cytokines such as IL-17 and IFN-γ is increased [61]. These molecules increase the permeability of blood vessels in the ovary, allowing lymphocytes to enter ovarian stroma. Once the T cells enter the ovary, they accumulate around the growing follicles. CD8+ T cells directly attack granulosa cells that produce AMH. CD4+ T cells support inflammation that leads to ovarian fibrosis (replacement of functional tissue with connective tissue).

T cells and macrophages have receptors that recognize the Fc fragments of antibodies, which directs them to the ovarian tissue. If anti-ovarian antibodies are produced, they bind specifically to proteins on the surface of cells in the ovary, thus further marking the site of action of immune cells [62]. In healthy women, Tregs would stop this process at the beginning, but in lupus, this control mechanism is missing.

Tregs stop macrophages and T cells from entering the ovary by several mechanisms. Tregs produce powerful “calming” molecules, mainly IL-10 and TGF-β (transforming growth factor beta). These cytokines give a direct signal to macrophages to remain dormant and prevent the activation of effector T cells [63]. Tregs use “metabolic starvation” tactics—they are extremely effective in “stealing” resources. Tregs have numerous receptors for IL-2 (the signal that effector T cells need to multiply and attack). Tregs absorb all available IL-2 into the environment [64]. Without it, the invading T cells cannot be activated and die through apoptosis before they have damaged the follicles. Furthermore, Tregs directly inhibit antigen-presenting cells (APC, dendritic cells, or macrophages). In order for T cells to enter the ovary, they must first obtain permission from dendritic cells or macrophages. For this purpose, the Tregs use the CTLA-4 molecule, with which they bind to macrophages and dendritic cells. This interaction blocks the sending of activating signals from the APC to the T cells [65]. Tregs are also responsible for maintaining the vascular barrier in the ovary. By maintaining low levels of inflammation, they prevent endothelial cells from expressing adhesion molecules. Without these molecules, the T cells and macrophages that circulate in the blood simply transit past the ovary without being able to attach to the vessel wall and pass into the tissue [66]. Tregs are also responsible for the balance of possible macrophage actions. Macrophages have two faces: M1 (aggressive, destructive) and M2 (restorative, anti-inflammatory). Tregs release signals that force macrophages to become type M2 [67]. Instead of attacking the follicles and destroying granulosa cells, these macrophages begin to take care of tissue health and follicle growth. In lupus, Tregs are few or defective [68]. The “brake” of the inflammatory process is absent, the vessels become sticky, IL-2 remains available for invading aggressive lymphocytes, and macrophages activate their destructive M1 mode. This leads to massive infiltration of T cells and a drop in AMH.

## 4. What Antibodies Against Ovarian Tissue Are Synthesized in Lupus

**Figure 1:**
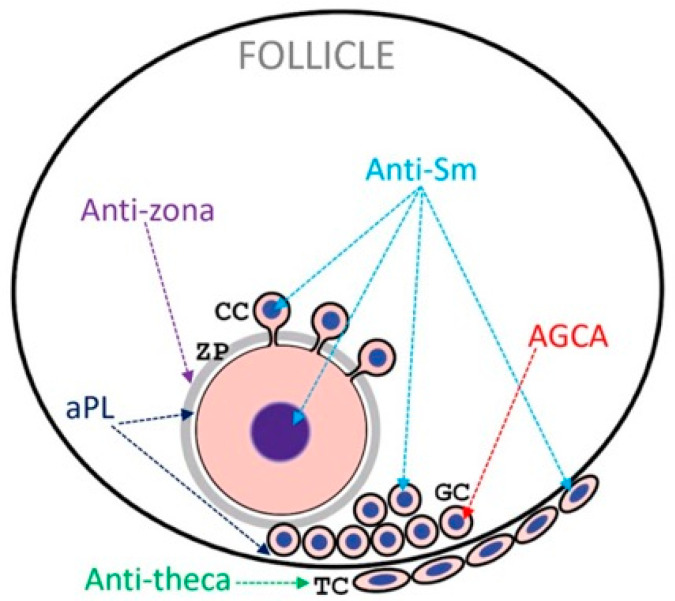
The figure presents the different types of autoantibodies that can negatively affect the function of different types of follicle cells during oogenesis.

Some types of autoantibodies synthesized in systemic lupus act specifically on ovarian tissue and are collectively referred to as anti-ovarian antibodies (AOAs). It has been proven that their presence is associated with disorders in reproductive function and premature depletion of ovarian reserve [69]. The main types of AOAs affecting oogenesis are of several types: antinuclear, anti-Sm antibodies, antibodies against granulosa cells, anti-theca cells, against the Zona pellucida (ZP), and antiphospholipid antibodies (Figure 1).

**Antinuclear antibodies (ANAs) are the most sensitive marker for lupus**. ANAs are considered the “gold standard” for systemic lupus screening because they are present in almost all patients with this disease [70]. Although they are very sensitive, they are not specific to lupus alone. A positive ANA test can also be observed in other autoimmune diseases (scleroderma, Sjögren’s syndrome, rheumatoid arthritis) [71]. About 5–15% of healthy individuals also have low ANA titers [72].

**Parts of ANA are designated as anti-Sm antibodies** (named after Stephanie Smith, the first patient in whom they were identified). Anti-Smith (anti-Sm) target small nuclear ribonucleoproteins (snRNPs) involved in mRNA splicing. They are present in 15–30% of patients with SLE and are rarely found in other autoimmune diseases. Therefore, these antibodies are considered highly specific biomarkers for systemic lupus [73]. Since anti-Sm antibodies are a marker for more severe forms of lupus, their presence often coincides with periods of high systemic inflammatory activity. Currently, the scientific consensus is that anti-Sm antibodies are more an indicator of a systemic autoimmune process that globally damages reproductive potential than a direct toxin for oocytes.

The direct molecular mechanism by which anti-Sm antibodies affect oocytes is not fully understood. It is assumed that the influence of anti-Sm is rather indirect and related to the general pathogenesis of systemic lupus [74]. Anti-Sm antibody target molecules (snRNPs) are present in all cells with a nucleus, including oocytes and ovarian granulosa cells. It has been suggested that when an inflammatory process is activated, these antibodies can cross the follicle barrier and trigger a local inflammatory response (oophoritis), which leads to the apoptosis of oocytes. This statement is based on the fact that immunoglobulins of the IgG class (anti-Sm belong to this class) are present in the follicle fluid [75]. Their concentration there is directly related to their serum levels, proving that they cross the blood–follicle barrier during follicle maturation. Studies have shown that patients with SLE have higher levels of anti-Sm antibodies in the follicle fluid, which alters the microenvironment of the oocyte. This can disturb the normal maturation of the oocyte and impair its ability to be fertilized and subsequently develop into an embryo [76]. The formation of immune complexes with the participation of anti-Sm in the systemic circulation and in the reproductive tissues themselves leads to the increased production of free radicals. This oxidative stress damages the cellular structures of the oocyte, including the mitochondria, which is critical for the maturation of the oocyte and early embryonic development.

A direct target of anti-Sm antibodies is a complex of proteins (B, B′, D1, D2, D3, E, F, and G) that are part of the spliceosome [77]. There is no evidence that pre-mRNA splicing is reduced by SLE. However, the process is seriously dysregulated—aberrant splicing events are increased [78]. This involves the preservation of introns in the final transcript, which often introduces premature stop codons and blocks the expression of affected genes. Splicing errors can create new amino acid sequences that the immune system can attack [79]. In the T cells of patients with lupus, insufficient production of normal forms of splicing regulators has been observed. This deficiency is due to the production of defective protein isoforms (e.g., abnormal forms of CD3ζ and RasGRP1) that impair the normal function of immune cells. Dysregulated splicing has been demonstrated in neutrophils, monocytes, and T-cells of lupus patients [80]. This often correlates with disease activity and specific symptoms such as lupus nephritis.

**Granulosa cell antibodies (AGCAs) contribute to impaired oogenesis**. The normal function of granulosa is critical for the development, differentiation, and maturation of the egg. Granulosa cells secrete estradiol and AMH and provide metabolic support to the developing oocyte. AGCAs bind to antigens on the surface of granulosa cells, resulting in their apoptosis [81]. This directly disturbs the oogenesis. The main membrane protein targeted by AGCAs is the follicle-stimulating hormone receptor (FSHR). Binding of antibodies to this receptor blocks the action of FSH and provokes the apoptosis of cells [82]. AGCAs also bind to luteinizing hormone receptors (LHRs) [83], as well as with other proteins, e.g., Ovarian Antigen-2 (OA-2). In normal situations, OA-2 acts as a specific surface marker that triggers the onset of degenerative processes in the follicles. Thus, OA-2 participates in the selection of a dominant follicle, marking those follicles that need to be eliminated [84].

**Anti-corpus luteum antibodies (Anti-CoL) lead to ovarian dysfunction through a direct autoimmune attack.** Their targets are specific structures responsible for producing hormones (mainly progesterone, but also of estradiol and inhibin A). Anti-CoL antibodies are found in about 15–22% of women with lupus. They are detected in the early stages of ovarian dysfunction and menstrual irregularities [85]. Anti-CoL antibodies recognize a specific 67 kDa glycoprotein, which is highly concentrated in the corpus luteum. This leads to local inflammation and damage to the cells of the corpus luteum. The presence of Anti-CoL antibodies is considered a specific marker of an autoimmune ovarian lesion and one of the causes of hypergonadotropic amenorrhea in lupus [45].

**Anti-theca antibodies are directed against antigens in theca interna**, the layer of cells surrounding the follicle. Theca cells synthesize androgens, which are precursors to estrogen, which is why theca antibodies are often classified as steroid cell antibodies (SCAs). The target of these antibodies is the enzyme 17-hydroxylase [86]. In cells, this enzyme converts pregnenolone and progesterone into DHEA and androstenedione. Antibodies against this enzyme block the production of estrogen in the ovary. This leads to a state of hypoestrogenism that causes a lack of ovulation, menstrual irregularities, and premature ovarian failure.

**Anti-zona antibodies bind directly to Zona pellucida**—the cell-free envelope made up of glycoproteins (ZP1, ZP2, ZP3) that surrounds the oocyte. Antibodies to these glycoproteins block their function—they interfere with the binding of spermatozoa to ZP and the activation of their acrosomal reaction, making fertilization impossible [87]. Levels of anti-ZP antibodies in patients with lupus are often high, which is associated with a more severe course of the autoimmune disease. Although they are not among the standard criteria for diagnosing lupus, they are a critical factor in planning a pregnancy in women with this disease.

**Antiphospholipid antibodies impair the membranes**. In lupus, antiphospholipid antibodies (aPL) are often observed. They are a specific type of autoantibodies against their own phospholipids or proteins associated with them. Their presence is a major sign of antiphospholipid syndrome (APS), a condition that increases the risk of blood clots and reproductive problems [88]. aPL are found in the follicle fluid [89]. Their action, on the one hand, changes the behavior of immune cells, and on the other hand, damages the blood vessels in the ovary. The most common target molecule of aPL antibodies is β2-glycoprotein I (β2GPI), which is found on the surface of granulosa cells and the oocyte itself [90].

Upon contact with cell membranes, aPL antibodies activate membrane receptors (such as TLR4 and Annexin A2) that send a signal inside the cells. The signal triggers the release of pro-inflammatory cytokines TNF-α and interleukins. It also provokes the secretion of tissue factor (TF), which is the main trigger of blood clotting. Thus, the inflammation is directly associated with thrombosis, which can damage the tissue [91]. The complement cascade is also activated, releasing fragments (such as C5a). They additionally attract and activate neutrophils and monocytes, intensifying local inflammation [92].

As a result of contact with aPL antibodies, cells produce proteins that hold leukocytes to the vascular walls (E-selectin, VCAM-1, and ICAM-1) [93]. In addition, binding of antibodies inhibits nitric oxide synthesis, leading to vascular constriction and tissue dysfunction [80]. Antibodies interfere with the work of proteins such as Annexin V, which normally coats phospholipids and acts as a shield against clotting. When this shield is removed, thrombosis occurs, which leads to ischemia and disrupts tissue functions [94]. The action of aPL antibodies disrupts the function of membrane phospholipids such as cardiolipin and phosphatidylserine [90]. This disrupts the integrity of the membrane of the oocyte and its surrounding cells.

**The deposition of immune complexes in the ovaries can disrupt oogenesis** through several pathophysiological mechanisms that affect both the structure of the organ and its function. The process is provoked mostly by the failure to clear debris from apoptic and destroyed cells, which cause the synthesis of autoantibodies. Antigens (such as DNA fragments) bind to autoantibodies, forming soluble immune complexes. They are deposited on the walls of small blood vessels in the ovary, causing ovarian vasculitis [95]. The accumulated immune complexes activate the complement system (in particular, the C3 and C4 proteins). This leads to the release of pro-inflammatory molecules (C3a, C3b, C4a). Inflammation can lead to the formation of microthrombosis, vascular occlusion, and hemorrhages. Thus, it deprives the ovarian tissue of oxygen and leads to degeneration of the follicles. In addition, through chemotaxis, pro-inflammatory molecules attract neutrophils to the vascular walls, which secrete lysing enzymes (such as collagenase and elastase). These directly attack the vascular walls, causing necrosis [96].

## 5. How Lupus Disturbs Oocyte Meiosis

**Figure 2:**
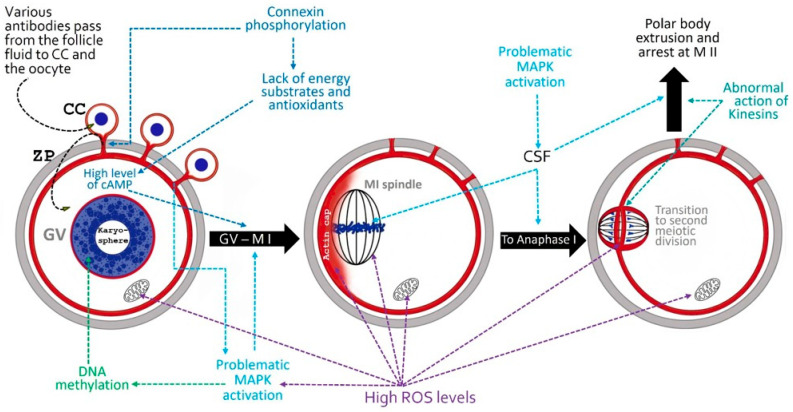
The figure presents processes that can disrupt the proper course of oocyte meiosis and the arrangement of meiotic structures. Blue structures—chromosomes; red layer—fibrillar actin. The left figure represents an oocyte with a formed karyosphere. At this stage, the nucleolus is transformed into a spherical structure surrounded by heterochromatin (karyosphere, surrounded nucleolus). When the karyosphere is formed, the oocyte has reached its maximal size. The follicle is in the stage of a tertiary (antral) follicle, just before its transformation into a Graafian (preovulatory) follicle. The central and right figures represent oocytes in preovulatory follicles.

Systemic problems in immune homeostasis and inflammatory processes in the ovaries change the microenvironment in which oocytes develop and mature. As a result, systemic lupus leads to meiotic disorders in the oocytes in several directions (Figure 2).

**The course of meiosis is impaired by the dysregulation of cellular signaling pathways**. In lupus, signaling pathways (such as MAPK and TGF-β) are disrupted, leading to meiotic arrest. MAPK (Mitogen-Activated Protein Kinase) is the main trigger of meiosis. MAPK is required to maintain the activity of Maturation Promoting Factor (MPF, cyclin-dependent kinase complex 1 plus cyclin B). With impaired MAPK function, the required level of MPF is not reached, which prevents the cell from moving from prophase I to metaphase I.

Under normal conditions, meiosis in oocytes is regulated by the specific kinase Mos [97], which activates the MAPK pathway (in particular, its component Extracellular Signal-Regulated Kinases 1 and 2, ERK1/2) independently of external growth factors. These kinases have the task of transmitting the signal from the cell surface to the nucleus [98]. In lupus, the pro-inflammatory environment and autoantibodies can lead to reduced expression or activity of Mos, which directly blocks the activation of ERK1/2 and leads to meiotic arrest [99].

For proper meiotic maturation, double phosphorylation of MAPK (converting it to p-MAPK) is required. Studies on mouse models of lupus (such as MRL/lpr) have shown that with disease progression, p-MAPK levels in oocytes decrease significantly. This interferes with the assembly of the meiotic spindle and leads to meiotic arrest at metaphase I [100].

Systemic inflammation in SLE is also associated with changes in DNA methylation. IL-6 and TNF-alpha activate signaling pathways (e.g., STAT3) that directly increase the levels and activity of DNA methyltransferases [101]. Although the MAPK pathway in oocytes acts primarily at the translational level, changes in DNA methylation can affect the expression of regulatory molecules (such as RasGRP1) that underlie MAPK activation. When too many methyl groups accumulate in the promoter region of the RasGRP1 gene, the gene is turned off or suppressed. This results in low levels of RasGRP1. Conversely, reduced methylation can lead to the overexpression of RasGRP1. Overactive production of this protein can make immune cells overactive, which is often seen in autoimmune diseases such as systemic lupus [102].

**Lupus causes an accumulation of reactive oxygen species**. High levels of reactive oxygen species (ROS) can activate phosphatases (such as DUSPs) that dephosphorylate and inactivate MAPK, interrupting the signal chain necessary to complete meiosis [103,104]. In lupus, excessive ROS generation is due to changes in oxidative phosphorylation in mitochondria and to the action of cytosolic enzymes (NADPH-oxidase, NOX) during chronic inflammation [105]. Changes in oxidative phosphorylation lead to the “leakage” of electrons that convert oxygen into highly reactive free radicals. This damages cellular proteins and DNA. Chronic inflammation in the active phase of SLE can also lead to an overabundance of ROS, which causes DNA damage and cell necrosis, which in turn also boosts the immune response. Free radicals can alter the structure of the self-proteins, making them appear foreign to the immune system, provoking an autoimmune response. In addition, ROS stimulate the formation of neutrophil extracellular traps (NETs) [106]. NETs are the immune system’s defense mechanism—neutrophils shed their DNA in the form of a network to trap and destroy pathogens. When NETs are not cleared from the tissue, they give the immune system access to the nuclear material, leading to the production of antibodies (such as anti-dsDNA).

NOX is the key enzyme complex that links oxidative stress to epigenetic changes. The main function of NOX is to generate ROS. In the context of inflammation and methylation, it acts as a signal enhancer. The latest data on the link between NOX and systemic lupus reveal a complex picture: while chronic inflammation in tissues is often accompanied by elevated levels of ROS, genetic and functional studies have shown that deficiency (rather than overactivity) of the specific NOX2 complex (catalytic core of the NOX enzyme complex) is actually a major risk factor for developing the disease [107]. Several working groups have reported that NOX2 deficiency paradoxically exacerbates lupus symptoms in mouse models [108,109]. It has been suggested that reduced NOX activity interferes with the clearance of apoptotic cells and cell debris and enhances Toll-like receptor (TLR7/9) signaling, leading to a loss of immune tolerance against its own structures.

Abnormal NOX function in lupus patients could be due to a genetic predisposition. Some research has shown that the risk of developing lupus is increased in people carrying mutations in the genes for Neutrophil Cytosolic Factors NCF1 and NCF2. These are key components of the NOX enzyme complex, and their loss of function increases the risk of SLE [110].

**Impaired communications between oocytes and cumulus cells disrupt the MAPK signaling.** The MAPK signaling pathway in the oocyte also depends on paracrine signals from the surrounding cells. Those of them that tightly envelop the oocyte are referred to as cumulus cells. They are connected directly to the oocyte through cytoplasmic protrusions across ZP. Lupus damages the intercellular connections between the oocyte and cumulus cells (gap junctions). Impaired function of these intercellular contacts leads to disorders in oogenesis [111]—this hinders the influx of important regulators such as cAMP that modulate MAPK activity. The molecular mechanism of damage to intercellular junctions is due to chronic inflammation and oxidative stress, which alter the synthesis and function of key proteins. In autoimmune diseases such as lupus, inflammatory cytokines have been found to lead to a significant reduction in levels of Connexin 43, the main protein that makes up the channels between cells [112]. Low expression of Cx43 interrupts metabolic support to the oocyte. Systemic inflammation, disrupting signaling pathways (such as MAPK/ERK), stimulates abnormal phosphorylation of specific serine residues in Cx43. This leads to the closure or degradation of the channels, which interrupts the exchange of cAMP and cGMP between somatic cells and oocytes [113]. In addition, in chronic inflammation, connexins can go from the formation of functional gap junctions to the formation of hemichannels (connexons) [114]. In pathology and stress, the connection of hemichannels in pairs is problematic. Thus, the hemichannels open directly to the intercellular space. This leads to the release of ATP and pro-inflammatory cytokines into the extracellular space, which further deteriorates the microenvironment of the follicle. A vicious circle is formed: the leaked ATP outside the cell acts as a signal for even stronger inflammation, which opens more hemichannels. Due to damaged connections with cumulus cells, the oocyte does not receive enough energy substrates (such as pyruvate) and antioxidants. This leads to mitochondrial dysfunction in the oocyte itself and the arrest of its development.

**Impaired communication between oocytes and cumulus cells disrupts the first meiotic arrest**. Maintaining high levels of cAMP in the oocyte is known to keep meiosis “on pause” in the first meiotic arrest. Stopping meiosis in prophase I is achieved by maintaining a high level of cAMP in the follicle fluid, which in turn maintains the activity of Phosphokinase A (PKA) [115]. In this situation, the oocyte remains with an intact nucleus, which is traditionally called the germinal vesicle. Impaired gap junction function leads to a premature transition from prophase I to subsequent meiotic phases.

**Impaired function of cytostatic factor disrupts the second meiotic arrest.** MAPK is involved in the formation of cytostatic factor (CSF), which keeps the oocyte arrested in metaphase II until the moment of fertilization. CSF holds the oocyte in a stable resting state for hours or even days, ensuring that it is ready and competent for fertilization. CSF inhibits the breakdown of proteins that are necessary to maintain the structure of the metaphase spindle. Specifically, CSF prevents the activation of the APC/C (Anaphase-Promoting Complex/Cyclosome) complex, which would otherwise end metaphase [116]. If there is a contact between the cell membranes of the oocyte and the sperm, calcium ions are released from the endoplasmic reticulum into the cytoplasm of the oocyte. The ions activate enzymes that destroy the CSF, allowing meiosis to complete and zygote division to begin. In addition, CSF keeps the MPF levels high [117]. This keeps the chromosomes arranged at the equator of the meiotic spindle (metaphase plate) in anticipation of the spermatozoon. In other words, MPF is the engine of cell division, and CSF is the handbrake that keeps it at high speeds, but in one place until the time comes for fertilization. When there is contact with a spermatrozoon, the calcium signal inactivates CSF. Without its protection, MPF breaks down instantly and the meiosis of the oocyte from metaphase II passes into anaphase II and telophase II, after which the second polar body is extruded—the oocyte is turned into a zygote. The participation of MAPK in this process is carried out through the following signal chain. It starts with the synthesis of the Mos protein (MAPKK, MAPK kinase kinase) that activates MEK1, which in turn activates MAPK. MAPK activates the p90Rsk kinase. This kinase phosphorylates the protein Emi2 (also known as Erp1), which stabilizes Emi2 and increases its activity. Stabilized Emi2 binds to APC/C (anaphase-promoting complex/cyclosome) and blocks its function. Since APC/C is responsible for the breakdown of cyclin B, its blocking maintains high levels of MPF [118].

## 6. Mouse Models Used to Study Oogenesis in Lupus Conditions

Several mouse models have been used to study ovogenesis and reproductive functions in systemic lupus. Each of them mimics different aspects of human disease and its impact on the ovaries. Two mouse models were based on genetic features of the mouse lines that led to the spontaneous development of lupus. The most commonly used model for the study of ovarian failure in lupus is the MRL/lpr (Murphy Roths Large) line [119,120]. These mice develop severe autoimmune syndrome. In them, as the disease progresses, there is a reduced number of antral follicles and impaired ovarian morphology, increased infiltration of T-cells and inflammatory factors in the ovarian tissue, and impaired quality of oocytes [100,121]. The other widely used model is NZB/W F1 (New Zealand Black/White), which is based on the spontaneous development of lupus in mice [122]. In these mice, as in humans, the disease mainly affects female individuals. The model is used to study the relationship between estrogen, autoimmunity, and reproductive health. Research has shown a gradual decrease in fertility in parallel with the development of lupus nephritis.

Another model used mice in which the disease is artificially induced. This allows changes to be tracked in ovogenesis from the very beginning of pathology. Such a lupus model is a Pristane-induced model based on Balb/c mice [123]. The mechanism by which the mineral oil Pristane (2,6,10,14-tetramethylpentadecane) induces lupus in Balb/c mice involves activating specific immune pathways that mimic the symptoms of human lupus. Intraperitoneal injection triggers strong inflammatory response in the abdominal cavity [124]. Oil droplets are phagocytized by monocytes, resulting in the formation of lipogranulomas and ectopic lymphoid tissue, where autoantibodies are produced. This model is characterized by the increased production of type I interferon (this mainly activates immature monocytes) and the activation of Toll-like receptors. In addition, Pristane induces apoptosis and necrosis. As a result, intracellular structures (such as small nuclear ribonucleoproteins) are released into the intercellular space, triggering an immune response [125]. Studies in this model, as well as in the MRL/lpr line, revealed defects in the cytoskeleton and chromatin structures (chromosomal condensation and alignment) of oocytes [100,126].

For lupus research, the BXSB model is also used, in which lupus also develops spontaneously. This model is atypical in that the disease is more severe in males due to a mutation in the Y-chromosome (Yaa gene, Y-linked autoimmune acceleration). The mutation is a translocation of a segment of the X chromosome to the Y chromosome, resulting in a duplication of the Tlr7 gene [127]. The BXSB model is key to understanding genetic factors and the role of TLR7 in disease development.

While mice offer a tractable way to study autoimmune-driven oocyte damage, they differ from humans in timeline, ovarian anatomy, and immune signaling.

− Human oocytes can remain in prophase I meiotic arrest for up to 50 years, whereas in mice, this period is only 4–6 weeks. This means that human oocytes have a vastly longer exposure window to chronic inflammation and autoantibodies characteristic of lupus.
− Mouse reproductive aging is accelerated; a 9–12 month old mouse approximates a human in their late 30s to mid-40s regarding ovarian reserve and function [128].
− Humans are typically mono-ovulatory, while mice sustain a larger pool of growing follicles to support multiple ovulations and large litters. This may dilute or concentrate the local effects of inflammatory cytokines differently between species [128].
− Differences are also observed in the manifestation of the disease. While lupus-prone mice (like the MRL/lpr or pristane-induced models) develop autoantibodies and glomerulonephritis, they often lack other human clinical features like rashes or arthritis [129].

## 7. How Lupus Damages Oocyte Structures

Systemic lupus can directly and indirectly affect the quality of oocytes and their development. While many women with lupus have successful pregnancies, the disease and its treatment can lead to structural and functional changes in reproductive cells. The direct influence of the disease leads to a decrease in the quality of oocytes. This is most directly illustrated by defects in oocyte structures. Studies in animal models have shown that the abnormal immune processes characteristic of lupus damage the organization of the oocyte cytoskeleton, mitochondria, endoplasmic reticulum, chromosomal condensation, and chromosomal alignment meiotic spindle.

The molecular causes of the abnormal arrangement of microtubules in the meiotic spindle are associated with chronic inflammation and oxidative stress, which directly damage cellular processes.

**ROS disrupt microtubule stabilization**. Inflammation induced by SLE leads to the increased production of ROS in the mitochondria of the oocyte. ROS disrupt the stabilization of microtubules through several direct and indirect biochemical mechanisms that lead to their depolymerization and structural damage. ROS oxidize specific cysteine residues in tubulin dimers [130]. This chemical alteration reduces the ability of the tubulin to polymerize properly. Cys12 and Cys354 of the tubulin molecule have been found to be among the most reactive sites [131]. The two cysteine residues, Cys12 and Cys354, are located in the β subunit of tubulin. Cys12 is located near the GTP binding site [132]. Cys354 is located in the Colchicine Binding Site [133]. This is why their chemical modifications disrupt the function of microtubules.

Elevated ROS levels affect the EB1 protein (end-binding protein 1), which stabilizes the growing ends of microtubules [134]. EB1 has a high affinity for tubulin intermediate states (GTP-tubulin and GDP-Pi-tubulin), which are found only at the (+) end of the growing microtubule. In this way, EB1 seals the dynamic end of the microtubule and prevents it from disintegrating. Oxidative stress impairs the function of EB1, leading to defects in the growth of microtubules and their stability.

ROS manipulate the activation of inappropriate signaling pathways dependent on cytoplasmic kinases (such as MAPs kinases). This leads to hyperphosphorylation of proteins key to the control of microtubules. When excessively phosphorylated, these proteins lose their ability to bind to microtubules and stabilize them. An example of this is the tau protein. Its function is best studied in neurons, where tau binds to microtubules and keeps them stable. Figuratively speaking, tau plays the role of sleepers that stabilize the parallel position of the microtubule rails. This protein has a connection to the function of microtubules in oocytes, although its role there is less understood. Studies on model organisms (such as *Xenopus*) have shown that tau, like its role in axons, helps stabilize microtubules in the meiotic spindle. Its role in ovogenesis is also evidenced by the fact that certain mutations in the tau gene interfere with the normal maturation of oocytes by destabilizing microtubules [135]. In oocytes, specific zones are observed in which tau mRNA is stored. This is further evidence of the connection between the protein tau and the cytoskeleton in the early stages of development.

**The stress in microtubules leads to stress in the actin cytoskeleton**. The breakdown of microtubules (provoked by ROS) releases specific factors (such as guanine nucleotide exchange factor-H1, GEF-H1) that activate the Rho pathways [136]. This causes further abnormal remodeling of the cytoskeleton. GEF-H1 acts as a molecular switch to regulate cellular architecture. Its action is unique in that it makes a connection between two main parts of the cytoskeleton—microtubules and actin microfilaments. In a normal situation, the GEF-H1 is firmly attached to the surface of the microtubules. As long as it is connected to them, it is catalytically inert and cannot interact with its targets [137]. The presence of ROS leads to depolymerization of the microtubule network, and GEF-H1 is released into the cytosol. Now free, GEF-H1 changes its conformation and becomes active. It acts as an exchange factor that replaces GDP with GTP on the small GTP-ase RhoA. The RhoA activated in this way drives the reorganization of the actin cytoskeleton. If RhoA is activated at the wrong time, inadequate behavior of the actin cytoskeleton can occur.

**The nucleation of microtubules is disturbed in lupus**. Mammalian oocytes lack centrosomes, and the spindle is formed in an unusual way. Instead of two compact centrioles, there are numerous scattered protein structures in the cytoplasm of the oocyte, designated as acentrosomal Microtubule-Organizing Centers (MTOCs). They initiate the growth of microtubules. The process is orchestrated by proteins localized on chromosomes. These proteins concentrate Ran-GTP around themselves [138]. Thus, a Ran-GTP gradient is created around the meiotic chromosomes. This gradient attracts microtubules near DNA. Motor proteins (such as kinesins and dynein) capture the newly formed microtubules and arrange them into a bipolar structure, forming the poles of the spindle. Impaired nucleation of microtubules directly affects the quality of the meiotic spindle.

Another important regulator of microtubule nucleation in mammalian oocytes is KIF20A (also known as MKlp2). It is a motor protein from the kinesin-6 family. Using ATP, it moves along the microtubules and thus participates in microtubule reorganization in the last stages of meiosis. The direct role of KIF20A is to deliver key factors (such as Aurora B) to the nucleation sites, ensuring the stability of the acentrosomal spindle [139]. In meiotic oocytes, the Aurora B kinase plays a key role as a conductor of microtubules. Aurora B is localized on chromosomes and stimulates the formation of microtubules directly around chromatin. It regulates proteins such as Stathmin and MCAK (Mitotic Centromere-Associated Kinesin), which normally destroy microtubules. By phosphorylating them, Aurora B suppresses their activity, allowing the spindle to stabilize around the chromosomes [140]. The most critical role of Aurora B is to monitor whether homologous chromosomes are properly attached to opposite spindle poles. If it receives a signal of improper connection between the kinetochores and microtubules, Aurora B cuts the connection, giving the spindle filaments a chance to attach properly to the chromosomal kinetochores. Thus, Aurora B helps to properly arrange the bivalents in the metaphase plate, ensuring the accurate separation of genetic material [141]. In short, with disruptions in the action of KIF20A, the action of Aurora is also disrupted, and as a result, the meiotic spindle of the oocyte remains disorganized, and the risk of aneuploidy increases dramatically.

**Abnormal action of KIF20A disturbs the asymmetric division of the oocyte.** The spindle in the first meiotic division is assembled in the center of the oocyte, and then it has to move to the periphery. At this spindle position, the separated polar body is very small, so that almost the entire cytoplasm remains in the oocyte. KIF20A helps maintain the integrity of the meiotic spindle during its migration to the oocyte cortex. As part of the Kinesin-6 family, KIF20A specifically stabilizes the overlapping zones of antiparallel microtubules in the middle of the spindle [142]. KIF20A functions as a homodimer. It possesses two motor domains that connect to microtubules. In the midzone, the motor simultaneously “steps” on two adjacent but oppositely oriented microtubules, connecting them physically [143]. This creates a structural support that allows the spindle to remain intact as it moves through the viscous cytoplasm pulled by the actin filaments. Furthermore, KIF20A directly interacts with the actomyosin filaments of the contractile ring during polar body detachment [144]. Therefore, if the action of KIF20A is disturbed, the spindle becomes disorganized, its movement to the cortex is problematic, the contractile ring cannot form properly, and the extrusion of the polar body fails. Thus, instead of one haploid set of chromosomes remaining in the oocyte, two remain. From the data presented, it can be seen that defects in microtubules and the organization of actin microfilaments are related by common molecular mechanisms. That is, the same factors can damage both elements of the oocytic cytoskeleton.

**Abnormal action of KIF20A also disturbs the polar body extrusion.** After the chromosomes divide during anaphase I, KIF20A is concentrated in the microtubule overlap zone between the two sets of chromosomes [145]. It organizes these microtubules into a stable bundle called the central spindle, which serves as a mechanical support in the formation of the contractile ring that is supposed to separate the polar body from the oocyte. KIF20A acts as a rail motor that delivers key proteins to the middle of the spindle. They form a complex known as Chromosomal Passenger Complex (CPC). This complex includes: Aurora B—the catalytic unit; INCENP (Inner Centromere Protein)—serves as a scaffold and binding link to the motor; Survivin—recognizes and binds directly to the centromeres of chromosomes and signals in case of improper attachment of chromosomes to microtubules; Borealin—directs the complex to the middle of the spindle during cytokinesis [146,147]. It can be summarized that in mammalian oocytes, KIF20A is absolutely critical for cytokinesis and the successful extrusion of the polar body.

## 8. Direct Evidence of Cytoskeletal Damages in the Oocytes in Lupus Mice

In oocytes derived from mouse models with lupus, severe defects in the morphology of actin and tubulin structures (the meiotic spindle and the associated actin cap) are observed. Since the interaction between actin microfilaments and microtubules is critical for spindle positioning and polar body extrusion, these damages directly degrade the quality of the oocyte. This has been observed in both the spontaneous development of lupus [89,90,91,92,93,94,95,96,97,98,99,100] and artificially induced lupus [126]. In addition, defects in the organization of microtubules and actin filaments interfere with the proper rearrangement of all organelles during the different phases of meiosis. In the Pristane model, abnormal spindles in metaphase I (MI) of meiosis were observed in about 60% of the studied oocytes. Defects include the disorientation of microtubules, too large spindle, and too wide spindle poles. In the MRL/lpr lineage, the proportion of defective spindles exceeds 90%. The structure of the actin caps of meiotic spindles in MI in lupus mice also shows defects. They are observed in about 50% of oocytes in the Pristane model [126]. In the MRL/lpr line, the percentage of normal aitin caps in MI is about 30% [100]. Defects in the actin cytoskeleton include abnormally small diameter of the actin cap, uneven distribution of its actin, or a complete absence of an actin cap. In addition, in these two models, a deterioration in the quality of chromosomes in meiotic oocytes was observed—the presence of not completely compact chromosomes, as well as visibly degenerated chromosomes was reported. In mice that spontaneously develop lupus, chromosomal condensation in MI is incomplete in about half of the oocytes. Lowest level of normal chromosomal condensation was found in young lupus mice (7 weeks of age)—less than 1/3 of the oocytes had normally condensed chromosomes. However, the rate of chromosomal degradation is highest among adult (at 20 weeks of age) postpartum mice. This suggests that in the group of young mice, there is a problem with the condensation rate, which does not lead to catastrophic failure and chromosomal degradation, while in the group of old postpartum mice, the eggs tend to mature on an all-or-nothing basis.

**DNA damage leads to defects in the oocyte cytoskeleton**. The increased level of ROS, characteristic of lupus, causes direct damage to the DNA of oocytes. The most common direct damage is the oxidation of guanine, which produces 8-oxodG (8-Oxo-7,8-dihydro-2′-deoxyguanosine) [148]. The presence of this abnormal base leads to mutations during replication. The telomeres of chromosomes are extremely rich in guanine and are therefore an easy target of free radicals. Oxidative stress shortens telomeres prematurely, leading to cellular aging of the oocyte [149]. ROS can directly break down the sugar-phosphate backbone of DNA [150]. Double-stranded breaks are the most dangerous because they are difficult to repair and often lead to the loss of chromosomal segments [151]. In oocytes, these damages are critical because they have a limited capacity to repair DNA [152]. Free radicals can irreversibly bind the DNA strand to neighboring proteins [153]. This blocks both replication and transcription. The accumulation of immune complexes in the ovaries maintains local inflammation, which stimulates the formation of ROS, and this further worsens the quality of DNA in conditions of lupus.

**Lupus-specific antibodies contribute to defects of the oocytic cytoskeleton**. The presence of autoantibodies can directly affect the quality of oocytes and induce apoptotic pathways that disorganize the meiotic apparatus. Lupus-specific autoantibodies can enter cells and bind to intracellular targets. For example, anti-dsDNA antibodies have been found to have the ability to cross the cell membrane of living cells [154], where they induce apoptosis [155]. The cited research changes the classical immunological dogma that the inside of the cell is inaccessible to antibodies. In patients with lupus, the presence of these antibodies in the microenvironment of the follicle and their penetration into the oocyte directly affects reproductive outcomes. For example, anti-Ro/SSA and anti-La/SSB autoantibodies are found in the follicle fluid, from where they can penetrate the oocyte [156]. We can suppose that these antibodies from the follicle fluid pass into the cumulus cells, and then through the cytoplasmic bridges between the cumulus cells and the oocyte, the antibodies can enter the ooplasm. Anti-Ro/SSA blocks the action of the Ro52 protein, which controls the ubiquitination of damaged proteins; anti-La/SSB antibodies block Ro60, which is responsible for the destruction of misfolded RNA.

It has been proven that anti-tubulin antibodies (anti-tubulin-α-1C) are also produced in lupus. Their level correlates with the activity of the disease and specific clinical manifestations such as cutaneous and mucous vasculitis [157]. To date, there is no conclusive scientific evidence that anti-tubulin-α-1C directly penetrates the oocyte to bind to the meiotic spindle. So far, it has been accepted that their influence on meiotic cytoskeletal structures is indirect. There is also no valid evidence that anti-actin antibodies can penetrate living cells, including oocytes. However, there is evidence that antibodies against microfilaments are found in the cytoplasm of oocytes [158]. Antibodies to actinin (a protein that binds actin filaments to each other) are found in about 20% of patients with SLE. The presence of these antibodies often correlate with anti-dsDNA antibody levels, suggesting that some anti-DNA antibodies may directly bind to actinin [159].

**In systemic lupus, the function of mitochondria in oocytes is impaired**. Oxidative stress in oocytes causes direct damage to mitochondrial DNA (by a mechanism that was explained above) and disrupts the integrity of mitochondrial membranes. Studies have shown significantly higher rates of lesions in mtDNA and a reduced number of mtDNA copies in patients with SLE compared to healthy people [160]. Oxidized mtDNA (ox-mtDNA) does not break down properly and is released by neutrophils as a powerful antigen that stimulates the production of interferon-α and the formation of anti-mtDNA antibodies [161]. ROS also directly damage the structural components of mitochondrial membranes. Highly active oxygen radicals attack polyunsaturated fatty acids. This leads to the formation of toxic products such as malondialdehyde (MDA) and 4-hydroxynonenal (HNE), which alter membrane permeability and fluidity [162]. The oxidative modification of membrane proteins disrupts the functioning of the electron transfer chain, which creates a vicious circle—damaged membranes generate even more ROS. Lupus inhibits the creation of new mitochondria and the mitophagy of damaged mitochondria. In the T cells of patients with lupus, an overexpression of the RAB4A protein is observed, which interferes with the fusion of autophagosomes with lysosomes [163]. This blocks the breakdown of damaged mitochondria. Accumulated damaged mitochondria release mitochondrial DNA and ROS into the cytosol. These molecules act as powerful inflammatory signals, activating the production of interferon-α [149,150,151,152,153,154,155,156,157,158,159,160,161,162,163]. The problem of mitochondrial renewal and impaired ATP production is one of the main causes of reproductive difficulties in women with lupus, even with normal ovarian reserve.

It was observed that a specific type of autoantibodies directly attack the mitochondrial components. They are referred to as antimitochondrial autoantibodies (AMAs) [164]. These antibodies target different structures in the mitochondria. In lupus patients, the following are described: anti-M2 antibodies directed to pyruvate dehydrogenase complex; antibodies against mitochondrial DNA (anti-mtDNA), which bind directly to the genetic material of mitochondria; and antibodies against proteins of the outer mitochondrial membrane [164]. In addition, other autoantibodies attacking cell structures can activate signaling pathways that lead to mitochondrial dysfunction.

**In systemic lupus, the function of the endoplasmic reticulum (ER) in oocytes is impaired.** A combination of autoimmune, inflammatory, and metabolic factors contributes to this [165]. The constant presence of inflammatory cytokines in the follicle fluid disrupts ER homeostasis. ER is the main depot for calcium in the oocyte. Lupus can cause calcium deficiency or improper release, which interferes with the proper folding of proteins. Under stress, proteins with a defective structure accumulate in the lumen of the ER, which activates a process called Unfolded Protein Response (UPR) [166]. One of the main UPR transducers is the PERK protinkinase (Protein kinase RNA-like ER kinase). Under normal conditions, the lumenal domain of PERK is bound to the Grp78 protein and thus remains inactive. Under stress, PERK is released by Grp78 PERK and acts as a chaperone that helps to fold proteins properly [167]. This leads to a temporary suspension of total protein translation. The goal is to reduce the incoming flow of new proteins into the ER. If the UPR fails to restore balance, the CHOP PROTEIN (C/EBP homologous protein) is activated. It reduces levels of the anti-apoptotic protein Bcl-2, making both cumulus cells and oocytes more prone to apoptosis. Due to its role in cell death, CHOP has been linked to the development of other autoimmune diseases, as well as neurodegenerative diseases and certain cancers, in addition to lupus [168].

## 9. How Lupus Affects Early Embryonic Development

SLE affects reproductive ability not only by reducing ovarian reserve, but also by disrupting the processes necessary for the development of a viable embryo. As explained above, patients with lupus have lower levels of AMH. In the ovaries, this hormone acts as the main inhibitor, which prevents premature depletion of ovarian reserve. AMH inhibits the transition of follicles from primordial (dormant) to primary stage. It reduces sensitivity to FSH by reducing the expression of FSH receptors. It also inhibits the enzyme aromatase, which limits the production of estrogens. The molecular mechanism is as follows: AMH binds to its receptor AMHRII, which activates SMAD proteins (SMAD1, 5, and 8) [169]. These proteins enter the nucleus of cells and alter the transcription of genes responsible for cell growth and differentiation. By keeping the FSH sensitivity levels under control, AMH helps select only one dominant follicle, preventing too many oocytes from maturing at the same time. Thus, a normal level of AMH ensures that the oocytes mature in a controlled manner and in small portions. In in vitro fertilization procedures, it has been found that patients with lower levels of AMH produce fewer embryos of good quality at the cleavage stage and reduce the chance of embryos reaching the blastocyst stage [170].

## 10. Conclusions

We can summarize that oocyte damage in systemic lupus erythematosus is a complex process that affects oocyte quality and reproductive potential. During meiosis, the delicate process of division in which the genetic material of the future embryo is formed, the structures of the oocyte are under attack from several main directions. (1) The systemic and local inflammation characteristic of lupus affects the microenvironment of the ovary. High levels of pro-inflammatory cytokines in the follicle fluid lead to damage to meiotic cytoskeletal rearrangements in oocytes and to the assembly of a properly functioning meiotic spindle. The accumulation of free radicals damages both the cell membranes of follicle cells and oocytes, as well as mitochondrial membranes. Impaired function of oocytic mitochondria leads to energy starvation and abnormal meiosis. (2) The presence of specific autoantibodies can directly attack the structures of the ovary. Different types of anti-ovarian antibodies can bind to components of the oocyte or follicle cells that feed it, causing premature apoptosis. Antiphospholipid antibodies impair blood supply to follicles, leading to ischemia and nutrient deficiencies for meiotic division. (3) Clinical manifestations of lupus lead to genetic instability. Meiosis requires precise arrangement of chromosomes in the meiotic spindle. The inflammatory environment interferes with the proper assembly of microtubules. This increases the risk of aneuploidy in oocytes and future embryos.

## Data Availability

For the preparation of this review, no new data were created.
